# Performance Comparison of Cross-Like Hall Plates with Different Covering Layers

**DOI:** 10.3390/s150100672

**Published:** 2014-12-31

**Authors:** Fei Lyu, Zhenyan Zhang, Eng-Huat Toh, Xinfu Liu, Yinjie Ding, Yifan Pan, Chengjie Li, Li Li, Jin Sha, Hongbing Pan

**Affiliations:** 1 School of Electronic Science & Engineering, Nanjing University, Nanjing 210093, China; E-Mails: lvfeieric@gmail.com (F.L.); zhangzhenyan518@163.com (Z.Z.); lichengjie.520@163.com (C.L.); lili@nju.edu.cn (L.L.); shajin@nju.edu.cn (J.S.); 2 GLOBALFOUNDRIES Singapore, Singapore 738406, Singapore; E-Mails: EngHuat.TOH@globalfoundries.com (E.-H.T.); XinFu.LIU@globalfoundries.com (X.L.); YINJIE.DING@globalfoundries.com (Y.D.); 3 Nanjing Foreign Language School, Nanjing 210008, China; E-Mail: panyifanhappy@163.com

**Keywords:** cross-like Hall plate, sensitivity, offset, P-type covering layer

## Abstract

This paper studies the effects of the covering layers on the performance of a cross-like Hall plate. Three different structures of a cross-like Hall plate in various sizes are designed and analyzed. The Hall plate sensitivity and offset are characterized using a self-built measurement system. The effect of the P-type region over the active area on the current-related sensitivity is studied for different Hall plate designs. In addition, the correlation between the P-type covering layer and offset is analyzed. The best structure out of three designs is determined. Besides, a modified eight-resistor circuit model for the Hall plate is presented with improved accuracy by taking the offset into account.

## 1. Introduction

Nowadays, magnetic sensors based on the Hall effect possess an increasingly wide application in the contact-less measurement for linear position, angular position, velocity, and so on [1–3]. The cross-like Hall plate provides an approach to realize high reliability, compact size, high accuracy and low cost.

As the important characteristics of a Hall sensor, both the current-related sensitivity and the offset have strong impacts on the performance of a Hall plate. A high sensitivity that improves the signal-to-noise ratio of the Hall sensor is often strongly limited by the short-circuit effect [4]. Another major limitation that affects Hall effect sensor performance is the offset. The possible reasons for the offset generation are related to the sensor fabrication process, packaging, operating conditions and aging [5]. Therefore, a real Hall sensor generally has a zero-field offset. In order to eliminate the offset of Hall sensors, a dynamic method known as the “current spinning technique” is often used [6–9]. The geometry plays an important role in the sensitivity and offset [10–15]. In the last few decades, Hall devices with various geometries had been studied, such as square, octagon, cross-like, *etc.* [16–18]. During these works, it is found that the most promising magnetic sensor with high sensitivity and a low offset is the cross-like Hall plate. For the according design of the signal conditioning circuit, an accurate circuit model for the Hall plate with offset is needed. Even though the existing circuit models for Hall plates are outstanding in some aspects, they more or less have a few disadvantages [19–22]. Some of them have even not obtained high accuracy. On the other side, others have not taken into account the essential physical effects, such as the temperature effect, parasitic effect, offset, and so on.

This work studies the current-related sensitivity and offset of a cross-like Hall plate with different covering layers in detail. We designed multiple Hall cells with different structures. Section 2 introduces these designs and the measurement data. Section 3 analyzes the measurement data from two major aspects: the current-related sensitivity and the offset. Moreover, a modified eight-resistor circuit model with improved accuracy is presented to help the sensor design. Section 4 summarizes all of the work and gives the conclusion.

## 2. Design and Experiment

### 2.1. Design of Hall Plate

In this study, we designed three different structures implemented in three Hall chips, and each chip contains five Hall cells in different sizes. All of the Hall chips are fabricated in 0.18-μm BCDlite^TM^ technology provided by GLOBALFOUNDRIES. Existing implants are employed to form the Hall devices without additional processes.

**Figure 1.**
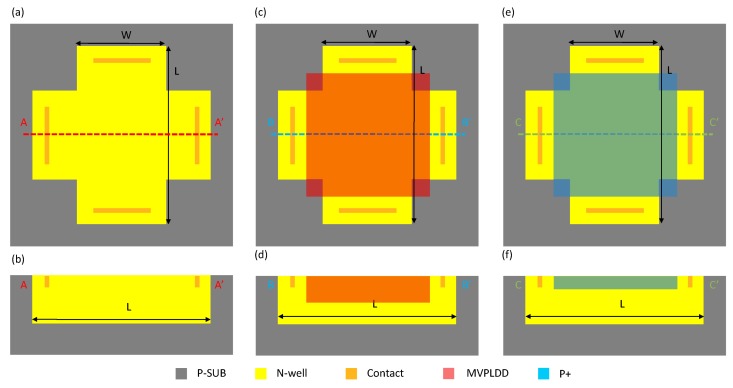
Top view (**a**) and cross-section (**b**) of the first structure (S1). Top view (**c**) and cross-section (**d**) of the second structure (S2). Top view (**e**) and cross-section (**f**) of the third structure (S3). L, length; w, width; P-SUB, P-type substrate; N-well, N-type well; MVPLDD, the drain extension of an LDPMOS.

**Figure 2.**
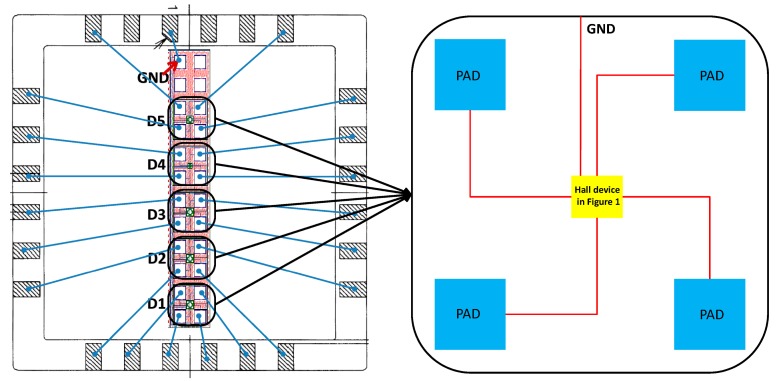
Schematic block diagram of Hall cells in a chip. PAD, contact pad; GND, the ground.

The top and sectional view of the first structure (S1) are shown in Figure 1a and Figure 1b, respectively. This structure consists of an N-well in a P-substrate and four contacts. The active area of the Hall plate is the N-type well region, which is formed in the step of the low voltage P-channel device substrate. The four contacts implemented with N+ implantation are 90° symmetrical. Additionally, two pairs of opposite contacts serve as biasing contacts and measurement contacts, respectively. Figure 1c and Figure 1d illustrate the top view and the cross-section of the second structure (S2). Compared to S1, the active region of the Hall plate is covered with MVPLDD, which is formed in the drain extension of an LDPMOS (laterally-diffused P-channel metal oxide semiconductor). The MVPLDD connected to the ground can generate a depletion region to protect the active region of the Hall plate from the interference of the upper surface. The third structure (S3) of the Hall plate only replaces the MVPLDD by the P+ implant to bury the active area of the Hall plate. In order to improve the stability of the Hall device, both the P+ and MVPLDD layers act as P-type layers to protect the active region of the Hall plate [18]. The schematic block diagram of Hall cells in a chip is shown in Figure 2.

### 2.2. Measurement

**Figure 3.**
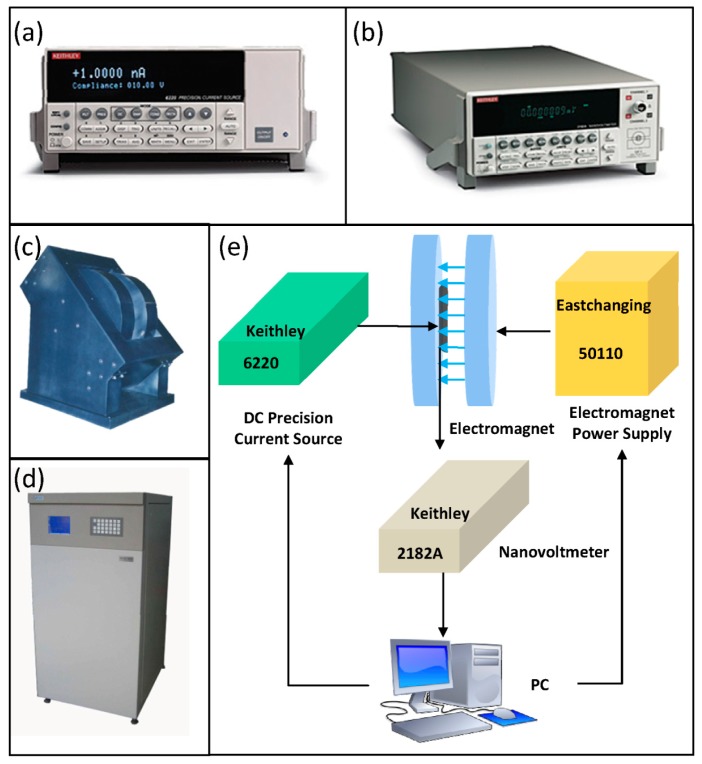
Measurement setup for Hall voltage: (**a**) Keithley 6220, a DC precision current source; (**b**) Keithley 2182A, a nanovoltmeter; (**c**) Electromagnet; (**d**) Eastchanging 50110, an electromagnet power-supplying equipment; (**e**) Whole measurement system.

**Figure 4.**
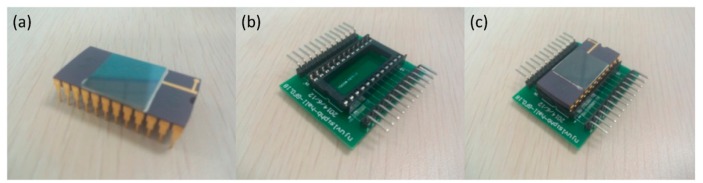
(**a**) Hall chip, (**b**) PCB (printed circuit board) and (**c**) Hall chip fixed on PCB.

**Figure 5.**
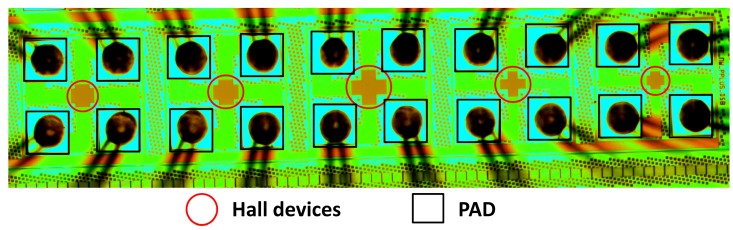
Microphotograph of the Hall chip.

**Figure 6.**
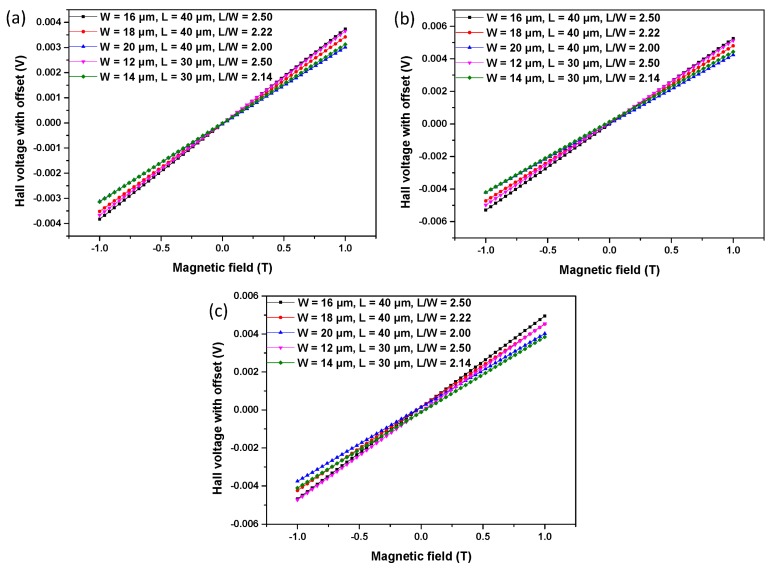
The measured Hall voltage (coupled with the offset) as a function of the applied magnetic field for S1 (**a**), S2 (**b**) and S3 (**c**) with different Ls and Ws.

The whole test system for Hall voltage is shown in Figure 3e. We use MATLAB to control other measurement components. The Keithley 6220 shown in Figure 3a, a DC precision current source, supplies a stable current to a Hall chip, which is shown in Figure 4, and the microphotograph of the Hall chip is shown in Figure 5. As an electromagnet power-supplying equipment, the Eastchanging 50110 in Figure 3d is used to generate a uniform magnetic field at the center of the electromagnet shown in Figure 3c. According to the principle of the Hall effect, the Hall voltage appears when the biased Hall chip is placed in the magnetic field. The Nanovoltmeter Keithley 2182A in Figure 3b can measure the values of the Hall voltage and send this information to a PC. All of the equipment runs at room temperature (∼30 °C). The biasing current is set at 0.1 mA. The value of the magnetic field varies from −1 T (tesla) to 1 T in 50 steps. Figure 6 illustrates three groups of the original test data. In order to avoid the influence from the residual magnetic field on the offset voltage, the Hall chips are taken away from the center of the electromagnet. The Keithley 6220 and Keithley 2182A are also used as the DC precision current source to supply a stable current for Hall cells and the nanovoltmeter to measure the offset voltage. Currents of 0.02 mA, 0.04 mA, 0.06 mA, 0.08 mA and 0.1 mA are applied in sequence to bias the Hall cells, and the corresponding offset voltages are measured.

## 3. Results and Discussions

### 3.1. Current-Related Sensitivity

When a Hall device is biased with a current *I* and placed in an orthogonal magnetic field *B*, the Hall voltage *V_H_* appears between two measurement contacts. The Hall voltage *V_H_* is defined as:

(1) $$V_{H}=S_{I}IB$$

where *S_I_* is the current-related sensitivity. While the current-related sensitivity *S_I_* of Hall device has the following analytical expression [22]:

(2) $$S_{I}=\frac{Gr_{H}}{nqt_{\text{eff}}}$$

here, *G* is the geometrical correction factor, *r_H_* is the Hall factor, *t_eff_* is the effective thickness of the Hall device's active zone, *n* is the doping concentration of the active region of Hall plate and *q* is the elementary charge of an electron.

Generally, as an important parameter for current-related sensitivity, the geometrical correction factor *G* depends on not only the shape, but also the size of the Hall plate. For a cross-like Hall plate, the L/W (length/width) ratio works as the major influence on the geometrical correction factor of the Hall plate. The cross-like Hall plates with a high L/W ratio have been analyzed, and the geometrical correction factor *G* is expressed as follows [16]:

(3) $$G=1-5.0267\frac{\theta_{H}}{\tan\left(\theta_{H}\right)}e^{-\frac{\pi }{2}\frac{L}{W}}$$

If L/W ≥ 3.63, *G* of the above equation has an accuracy better than 0.5%; while the sizes of our Hall plates are not in this range. The correspondence among length (L), width (W), L/W, the structures and the current-related sensitivity is presented in Table 1. The current-related sensitivity with the same L is increased with the decreasing of W. In other words, the increase of the L/W ratio is beneficial to the current-related sensitivity for the Hall plate with the same L. However, if L and W are proportionally reduced, the value of current-related sensitivity also becomes smaller. Therefore, the current-related sensitivity not only depends on the L/W ratio, but also has a strong correlation with the value of the length and width. The larger length, width and L/W ratio lead to the improvement of the current-related sensitivity of the Hall plate.

**Figure 7.**
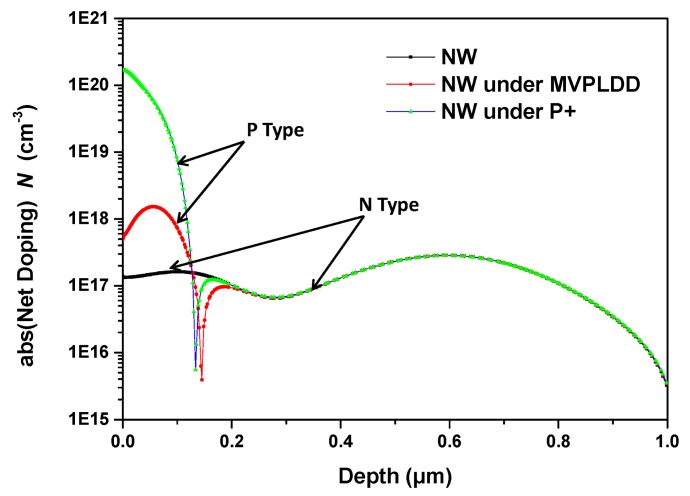
The net doping concentrations of the Hall plates for S1, S2 and S3 (provided by GLOBALFOUNDRIES).

On the other hand, both the doping concentration and the effective thickness of the Hall device's active region have a strong influence on the current-related sensitivity. The doping profiles of three structures are shown in Figure 7. The majority of the three curves in the N-type region coincide perfectly. Therefore, the depth of the N-type region can be used to estimate the current-related sensitivity. S2 shows the highest sensitivity among the three structures due to the smallest depth of the active region. Meanwhile, the areal concentration *nt_eff_* can be used to predicate the current-related sensitivity. The areal concentration is obtained by integrating the carrier concentration along the depth [23]:

(4) $$n=\int_{x_{b}}^{x_{l}}n\left(x\right)d\left(x\right)$$

where *n* is the three-dimensional concentration and *x_t_* and *x_b_* are the top and the bottom of the Hall plate's active region, respectively. Equation (4) shows that S2 also has the smallest three-dimensional concentration. The above analyses show an excellent agreement with the measurement data. In conclusion, considering the current-related sensitivity, the P-type doping area under the N-well is beneficial for the performance, and the MVPLDD is better than the P+ implant.

### 3.2. Offset

It is well know that the tested voltage *V_T_* contains two parts, the Hall voltage *V_H_* and the offset voltage *V_off_*. The tested voltage can be expressed by:

(5) $$V_{T}=V_{H}+V_{\text{off}}$$

**Figure 8.**
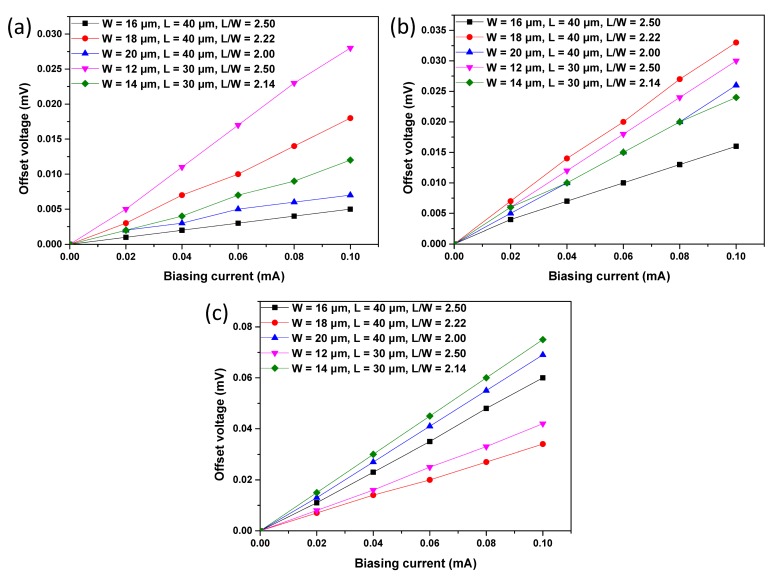
The measured offset voltage as a function of biasing current for S1 (**a**), S2 (**b**) and S3 (**c**) with different Ls and Ws.

Figure 8 displays the measured offset voltage at room temperature with respect to the biasing current of the three proposed structures. The offset voltage *V_offset_* is almost proportional to the biasing current *I_b_*. Therefore, we introduce a parameter *R_offset_*, which is defined as the following equation to express the value of the offset:

(6) $$R_{\text{offset}}=\frac{V_{\text{offset}}}{I_{b}}$$

The values of *R_offset_* of the three proposed structures are presented in Table 2. For S1, the Hall plate with a W of 16 μm and an L of 40 μm has the smallest *R_offset_* of 0.05 Ω. S2 with a W of 16 μm and an L of 40 μm shows the best performance in the aspect of offset. However, S3 with a W of 18 μm and an L of 40 μm performs best with the smallest offset. It is well known that the offset is influenced by several factors, while L and W are not the most important among them.

When the *R_offset_* is compared among the three structures, S1 is the best and S3 is the worst in the aspect of offset. Although the P-type doping region is necessary for the Hall plate to improve the stability, it increases the offset of the Hall plate. Both S2 and S3 have a lager offset than S1, with one more process on the active region of P-type doping. To our knowledge, the mask misalignment is a major cause of the offset [24]. As a result, the mask-misalignment that is inevitably introduced into the additional process increases the offset. On the other hand, the offset voltage is modulated by the PN junction depletion width at the P-layer and N-well, which also induces a more asymmetric contact region as a result of process variation. In addition, the P+ layer has higher doping than the MVPLDD, and the depletion width on the N-well side is larger than that of the MVPLDD side. As a result, the offset becomes worse, because the implants are not perfectly aligned and the dopant variation induces more asymmetry. Therefore, in order to have better stability and a lower offset voltage, the N-well under MVPLDD is preferred for the active region of the Hall plate.

### 3.3. Simulations by Silvaco TCAD

**Figure 9.**
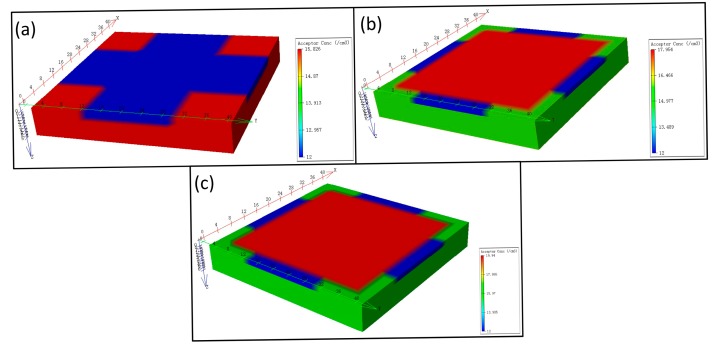
3D representation of S1 (**a**), S2 (**b**) and S3 (**c**).

To further assess the Hall effect sensor performance of the current-related sensitivity and offset, three different structures of Hall effect devices are modeled by Silvaco TCAD. The simulation tool helps in modeling the specific structures, to obtain the sensitivity and offset of the Hall plate. The simulation results provide useful information to verify the analysis of the experimental results. The three-dimensional representations of the three simulated structures are illustrated in Figure 9.

**Figure 10.**
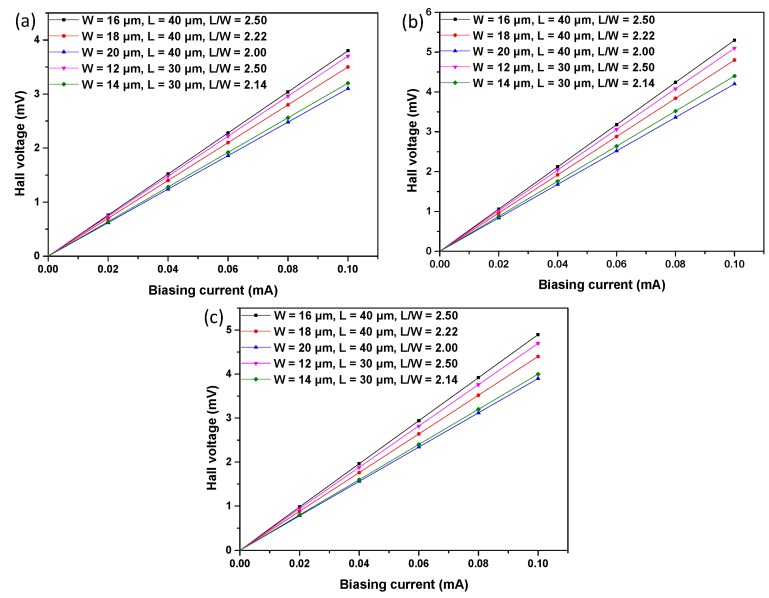
Simulation results of the Hall voltage of S1 (**a**), S2 (**b**) and S3 (**c**).

**Figure 11.**
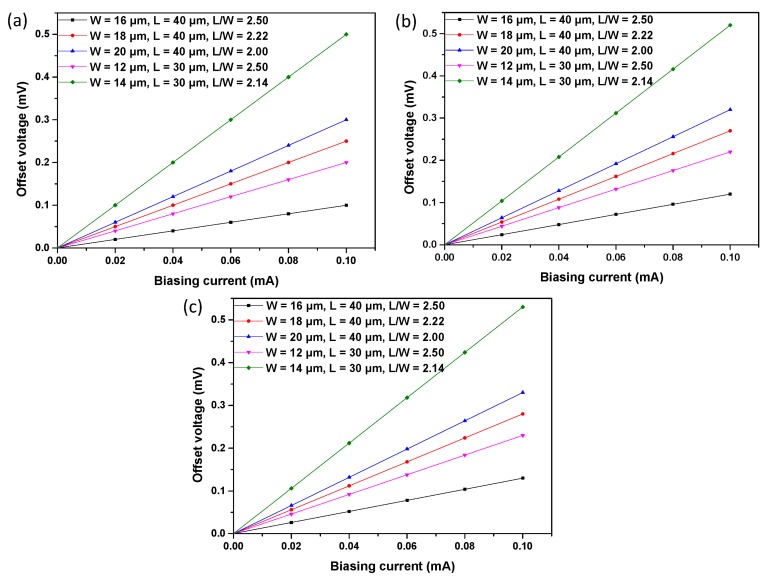
Simulation results for the offset voltage of S1 (**a**), S2 (**b**) and S3 (**c**).

For the analysis of current-related sensitivity, all of the structures were simulated using the biasing currents ramped from 0 to 1 mA, with a magnetic field of 1 T. Figure 10 shows the Hall voltage *versus* biasing current. Meanwhile, in order to simulate the offset, the asymmetry is introduced in the simulated structures by modify the size of the contact. Figure 11 illustrates the offset voltage with respect to the biasing current. Because of the packaging stress, heterogeneous doping concentration and technology process uncertainty, the simulation results and experiment results are not identical. However, the simulation results show the same tendency as the experiment results to demonstrate the correctness of the analysis of the experiment results.

### 3.4. Eight-Resistor Model with Offset

**Figure 12.**
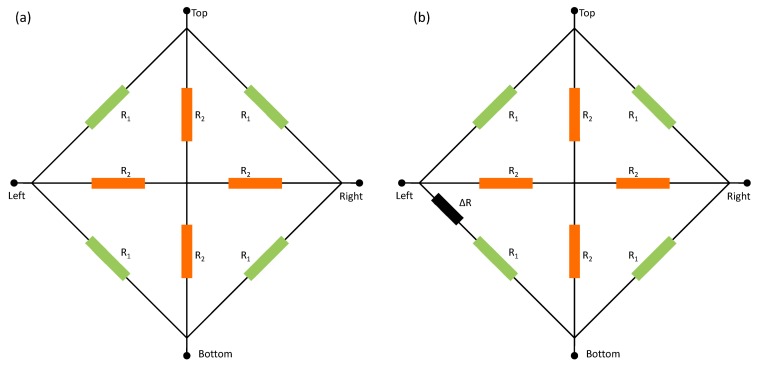
The measured offset voltage as a function of biasing current for S1 (**a**), S2 (**b**) and S3 (**c**) with different Ls and Ws.

In order to use the spinning current method to eliminate the offset in the signal conditioning circuit, a circuit model with offset is required in the design of the signal conditioning circuit [25–27]. A recent circuit model with eight non-linear resistors in Figure 12a can be used accurately and suitably for our proposed Hall plate [22]. This model covers the voltage-dependent non-linear effects, geometrical effects, temperature effects and packaging stress influences. In addition, it is relatively simple by only including a small number of physical and technological parameters. *R_1_* and *R_2_* are obtained by the symmetry of the Hall plate and the van der Pauw method of measuring the sheet resistance *Rs*. *R_1_* and *R_2_* can be expressed as:

(7) $$R_{1}=\left(\frac{2L}{W}-\frac{2}{3}-\frac{4\ln2}{\pi }\right)•R_{s}$$

(8) $$R_{1}/R_{2}=2-\frac{8}{\pi }\frac{\ln2}{L/W-1/3}$$

However, this model does not take the offset into account. We modified this model by taking the offset into account.

Because the relationship between offset voltage and biasing current is approximately linear, the offset is modeled by an offset resistor *ΔR*, as shown in Figure 12b. The right and left are used as biasing ports, and the top and bottom are defined for measurement. Without the offset resistor *ΔR*, when the circuit is biased by a current, the voltage difference between the top and bottom is zero. The offset resistor leads to the asymmetry of the Hall plate, which actually exists in the practical situation. Therefore, even though the model is without the influence of the magnetic field, an offset voltage still appears between the top and bottom due to the offset resistor, when the circuit is biased by a current. *ΔR* that we defined can be used to model the offset caused by all of the influence factors. By Kirchhoff's circuit law, *ΔR* can be calculated by:

(9) $$\Delta R=\frac{N}{M}R_{\text{offset}}$$

(10) $$N={R_{1}}^{4}+8{R_{1}}^{3}R_{2}+20{R_{1}}^{2}{R_{2}}^{2}+16R_{1}{R_{2}}^{3}$$

(11) $$M={R_{1}}^{2}{R_{2}}^{2}+4R_{1}{R_{2}}^{3}+{R_{1}}^{3}R_{\text{offset}}+6{R_{1}}^{2}R_{2}R_{\text{offset}}+10R_{1}{R_{2}}^{2}R_{\text{offset}}+4{R_{2}}^{3}R_{\text{offset}}$$

**Figure 13.**
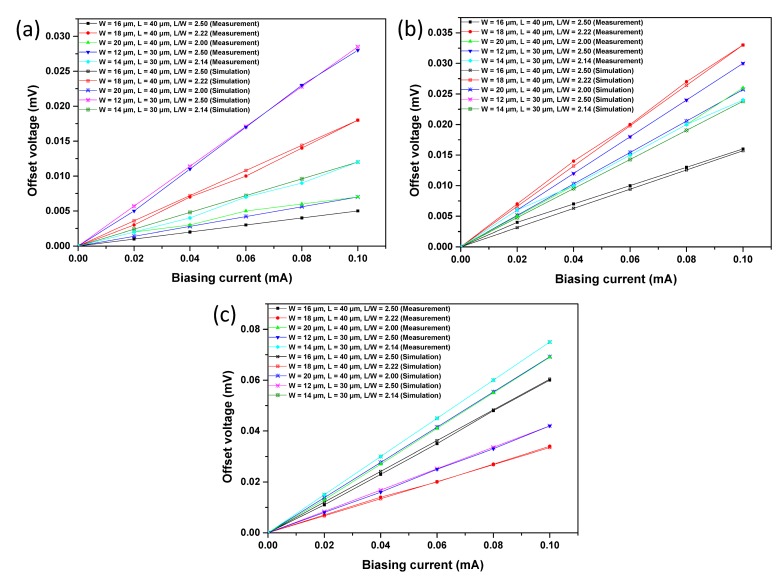
Comparisons between the measurements and the model simulation for the offset voltage.

The modified model has been written in the behavioral Verilog-A language and tested in a Cadence Spectre simulator. The modified eight-resistor model shows the same accuracy in sensitivity and temperature behavior as the model in [22], while the correctness and accuracy in the offset aspect are demonstrated by the corresponding experimental results of the Hall plate. A very good agreement with the model's simulation results is achieved and shown in Figure 13. Consequently, in SPICE-like (SPICE, simulation program with integrated circuit emphasis) EDA (electronic design automation) tools, the modified model can be used to do circuit simulation in the design of the signal conditioning circuit.

## 4. Conclusions

In order to analyze the current-related sensitivity and offset of the cross-like Hall plates with different covering layers, we designed and fabricated three structures of Hall plate in 0.18-μm BCDlite^TM^ technology provided by GLOBALFOUNDRIES. Each structure is implemented in a Hall chip with multiple Hall cells to study the impact of the L, W and L/W ratio on the performance of the Hall plate. Moreover, we demonstrate that the Hall plate with the MVPLDD layer above the active region is the best structure in both the current-related sensitivity and offset. Besides, we greatly improved the accuracy of the eight-resistor circuit model for the Hall plate by taking the offset into account.

## Figures and Tables

**Table 1.** Current-related sensitivity for Hall plates of different structures with respect to different W and L.

	**Sensitivity of S1 (V/(A·T))**	**Sensitivity of S2 (V/(A·T))**	**Sensitivity of S3 (V/(A·T))**
W = 16 μm, L = 40 μm, L/W = 2.50	37.8	52.7	48.1
W = 18μm, L = 40 μm, L/W = 2.22	34.7	47.6	43.8
W = 20 μm, L = 40 μm, L/W = 2.00	30.6	42.0	38.9
W = 12μm, L = 30 μm, L/W = 2.50	36.6	50.5	46.4
W = 14 μm, L = 30 μm, L/W = 2.14	31.3	43.1	39.7

**Table 2.** *R_offset_* for the Hall plates of different structures with respect to different W and L.

	***R****_offset_* **of S1(Ω)**	***R****_offset_* **of S2(Ω)**	***R****_offset_* **of S3(Ω)**
W = 16 μm, L = 40 μm, L/W = 2.50	0.050	0.157	0.604
W = 18 μm, L = 40 μm, L/W = 2.22	0.180	0.330	0.335
W = 20 μm, L = 40 μm, L/W = 2.00	0.070	0.257	0.692
W = 12 μm, L = 30 μm, L/W = 2.50	0.285	0.300	0.420
W = 14 μm, L = 30 μm, L/W = 2.14	0.120	0.238	0.750
